# Book Review: Social Ecology in the Digital Age: Solving Complex Problems in a Globalized World

**DOI:** 10.3389/fpsyg.2019.01579

**Published:** 2019-07-16

**Authors:** Rachel Kaplan, Jason Duvall

**Affiliations:** ^1^School for Environment and Sustainability, University of Michigan, Ann Arbor, MI, United States; ^2^Program in the Environment, University of Michigan, Ann Arbor, MI, United States

**Keywords:** cybersphere, multilevel research, translational, sustainability, sociocultural, transdisciplinary

With his astounding background, Daniel Stokols is uniquely qualified to author *Social Ecology in the Digital Age*, a sweeping, comprehensive work that invites readers to explore the history, core principles, and many applications of social ecology. Stokols is Research Professor and Chancellor's Professor Emeritus at the University of California, Irvine where he served as Director and Founding Dean of the School of Social Ecology. He holds appointments in the Departments of Psychology and Social Behavior, and Planning, Policy and Design, as well as in Public Health, Epidemiology and Nursing Science. In this book, Stokols touches on all these fields (and several others) to argue for the necessity and utility of social ecology. The social ecological perspective draws on concepts and approaches from the social sciences, as well as physical and life sciences and the humanities to examine the nested and interconnected nature of human-environment systems. In Stokols' view, this orientation and the emphasis that social ecology places on multidimensional and multilevel thinking are essential for confronting “the existential challenges of the twenty-first century.”

The book's 10 chapters embrace a more extensive range of topics than most readers would probably expect to find. In Chapter 1, Stokols describes his own “personal journey,” rooted in his childhood in Miami, Florida, and culminating with his decades of leadership at Irvine. The winding and often fortuitous path that Stokols documents reminds scholars of all ages about the importance of finding supportive settings that help us look beyond disciplinary boundaries. His own evolution as a social ecologist is reflected in the topics and examples in the rest of the book. The chapters detailing the historical origins (chapter 2) and core principles (chapter 3) of social ecology highlight a number of foundational studies that inform the outlook and methods of modern social ecologists. These early studies are also used to illustrate the interwoven complexities that often characterize human-environment transactions, a theme that is reiterated in later chapters, focusing on “promoting personal and public health” (chapter 5), “confronting complex social problems” (chapter 6), “managing global environmental change” (chapter 7), and “designing resilient and sustainable communities” (chapter 8).

In each of these chapters, Stokols provides a wide-ranging overview of the specific domain and outlines how the application of social ecology can help us better appreciate the myriad of environmental and socio-cultural factors at play. Carefully selected real-world problems, addressing such issues as environmental justice, educational programming, and climate policy, are incorporated to complement and enrich these discussions.

Two other chapters are especially noteworthy for their distinctive contributions. Chapter 4, “Rise of the Internet—Navigating Our Online and Place-based Ecologies,” offers much more than the title might suggest. In it, Stokols examines the “cybersphere” in environmental and ecological terms, showing parallels to more traditional perspectives of social and physical environments as well as ways in which the virtual environment directly and indirectly influences health, social behavior and environmental sustainability. Indeed, as Stokols depicts the cybersphere, it has fundamentally changed our social ecology in ways that could not have been anticipated. The importance of the cybersphere, and its far-reaching impacts, are further discussed within most of the subsequent chapters. In sum, these incisive discussions are a major, unique, and significant contribution. In fact, we feel that if this material were available as a separate monograph focused on the impact of the digital world from a social ecological perspective it would have an even greater chance of reaching receptive audiences.

The other chapter that we feel stands out for its noteworthy role is Chapter 9, “Educating the Next Generation of Social Ecologists.” Stokols' own career exemplifies his commitment to the organizing principles of this chapter, notably what he refers to as the “four T's”: transdisciplinary, team-based, translational, and transcultural perspectives. According to Stokols, these four T's are not only central elements of social ecological training, but are critical for tackling the environmental and social challenges that lie ahead. Adopting this orientation, however, can be challenging since it represents a significant departure from conventional disciplinary, and even cross-disciplinary, perspectives. Fortunately, Stokols offers readers practical advice about ways to cultivate the values, attitudes, and skills embraced by social ecology.

*Social Ecology in the Digital Age* is an invaluable contribution and a remarkable achievement. Its impressive breadth and depth, however, come with a downside. While *satisficing* (Simon, 1956) can be problematic, and “less is more” can lead to inappropriate minimalism, Stokols' approach may suffer from being at the other extreme. There are some significant costs to thoroughness. If one holds back on detail and (painfully) limits oneself to three or four main ideas, at least some of them are likely to be remembered. In our mind, that is preferable to leaving the reader overwhelmed with distinctions, categories, and nuances. As we all know from experience on both sides of the information-sharing divide, there is a great imbalance between the information provider's desire to share and the recipient's limited capacity to absorb and remember (Cowan, 2010). While we commend Stokols' use of concrete examples to help readers connect the dots, these stories are often interrupted by long passages of superfluous information. As a result, undergraduate students or those with only a passing interest in social ecology may find much of this volume difficult to access.

A similar tradeoff arises with respect to both the layout and the impressive collection of publications cited. In these times when we all rely on digital information and complain that attention has been hijacked by the fast-paced overstimulation provided by the cybersphere, it is important to consider how to present textual material. Using only half-inch margins may maximize words per page but decrease the likelihood that the words will be read. Likewise, the inclusion of more than 1,700 citations adds academic credibility, but the numeric listings make retrieval challenging.

*Social Ecology in the Digital Age* is extraordinary in its scope and depth. It brings together a wide-ranging set of ideas to argue for the power and promise of social ecology. In doing so, Stokols has crafted a meticulously constructed and comprehensive work that is likely to become requisite reading for serious scholars in a variety of fields related to social ecology. Hopefully broader audiences of professionals, policy-makers, and everyday citizens interested in social change will also turn to this font of insights because of its contribution in addressing many of “the existential challenges of the twenty-first century.”

## Author Contributions

Both authors listed have made a substantial, direct and intellectual contribution to the work, and approved it for publication.

### Conflict of Interest Statement

The authors declare that the research was conducted in the absence of any commercial or financial relationships that could be construed as a potential conflict of interest.
